# Taxonomy and Phylogeny of the *Fomitopsis pinicola* Complex With Descriptions of Six New Species From East Asia

**DOI:** 10.3389/fmicb.2021.644979

**Published:** 2021-03-26

**Authors:** Shun Liu, Mei-Ling Han, Tai-Min Xu, Yan Wang, Dong-Mei Wu, Bao-Kai Cui

**Affiliations:** ^1^Beijing Advanced Innovation Center for Tree Breeding by Molecular Design, Beijing Forestry University, Beijing, China; ^2^Institute of Microbiology, School of Ecology and Nature Conservation, Beijing Forestry University, Beijing, China; ^3^College of Life Sciences, Langfang Normal University, Langfang, China; ^4^Biotechnology Research Institute, Xinjiang Academy of Agricultural and Reclamation Sciences/Xinjiang Production and Construction Group Key Laboratory of Crop Germplasm Enhancement and Gene Resources Utilization, Shihezi, China

**Keywords:** brown-rot fungi, host specialization, multi-gene phylogeny, polypore, species complex

## Abstract

*Fomitopsis pinicola* is a common brown-rot fungal species found in northern hemisphere. It grows on many different gymnosperm and angiosperm trees. Recent studies show that it is a species complex; three species from North America and one species from Europe have been recognized in this complex. In the current study, six new species in the *Fomitopsis pinicola* complex were discovered from East Asia, based on morphological characters and phylogenetic analyses inferred from the sequence data of the internal transcribed spacer (ITS) regions, the second subunit of RNA polymerase II (RPB2), and the translation elongation factor 1-α gene (TEF). Detailed descriptions of the six new species are provided. Our results also indicates that species of the *F. pinicola* complex from East Asia usually have limited distribution areas and host specialization.

## Introduction

*Fomitopsis* P. Karst. was established by Karsten and typified by *F. pinicola* (Sw.) P. Karst. (Karsten, 1881). It is characterized by a perennial or annual growth habit, is sessile to effused-reflexed, has tough to woody hard basidiocarps, has a white to tan or pinkish-colored pore surface with mostly small and regular pores, has a dimitic to trimitic hyphal system with clamped generative hyphae, and has a hyaline, thin-walled, smooth, and ellipsoid to subglobose basidiospores which are negative in Melzer’s reagent; it causes a brown rot (Ryvarden and Johansen, 1980; Gilbertson and Ryvarden, 1986; Ryvarden and Gilbertson, 1993; Núñez and Ryvarden, 2001; Han et al., 2016).

*Fomitopsis pinicola* has been intensively studied because it has the function of dispelling wind-evil and dampness, and has anti-tumor (Dai et al., 2009; Sun et al., 2016), antifungal, antioxidant, immunomodulation, and neuroprotective activities (Guler et al., 2009; Bao et al., 2015; Sun et al., 2016; Guo and Wolf, 2018). Högberg et al. (1999) showed that all European populations of *F. pinicola* belong to one intersterility group. Ryvarden and Stokland (2008) described *Fomitopsis ochracea* Ryvarden & Stokland from Alberta on *Populus tremuloides* that was distinguished from *F. pinicola* by substrate preference, basidiospore morphology, and match flame test to the lacquered pilei surface. Subsequently, this species was proven to belong to the *F. pinicola* complex (Haight et al., 2019). Haight et al. (2016) suggested that *F. pinicola* is a species complex comprised of at least four well-supported phylogenetic species, three in North America (*F. ochracea* and two previously undescribed species) and one in Europe (*F. pinicola*). Haight et al. (2019) described two new species: *F. mounceae* Haight & Nakasone and *F. schrenkii* Haight & Nakasone from North America in the *F. pinicola* complex. Until now, four species have been recognized in the *F. pinicola* complex; *F. mounceae*, *F. ochracea*, and *F. schrenkii* from North America and *F. pinicola* from Europe.

In some cases, fungi species boundaries based on morphology misrepresents the number of existing species (Leavitt et al., 2011). Due to geographic isolation, lack of migration, and genetic drift there may be variation among populations, although this genetic variation is not always obvious (Haight et al., 2016). Cryptic species of species complexes are proving to be extremely common in higher fungi, particularly those with wide geographic distributions or host ranges (Haight et al., 2016); they share similar morphological characteristics and phylogenetic relationships to known species. More cryptic species could be discovered by combining evidence of morphological characters, molecular data, host trees, and distribution areas in species complexes (Liu et al., 2021).

In recent years, taxonomic and phylogenetic studies of *Fomitopsis* have been carried out in China and several new species have been described (Li et al., 2013; Han et al., 2014, 2016; Han and Cui, 2015; Liu et al., 2019), but none have been focused on the *F. pinicola* complex. Samples collected from China were still identified as *F. pinicola* complex in these studies. With more and more specimens collected from different areas of China, Vietnam, and of East Asia, six new species of the *F. pinicola* complex have been discovered based on morphological characters and phylogenetic analysis of ITS + RPB2 + TEF gene regions.

## Materials and Methods

### Taxa Sampling and Morphological Study

The examined specimens were deposited in the herbarium of the Institute of Microbiology, Beijing Forestry University (BJFC, Beijing, P. R. China). Morphological descriptions and abbreviations used in this study follow Han et al. (2016) and Liu et al. (2019).

### DNA Extraction and Molecular Analyses

The procedures for DNA extraction and polymerase chain reaction (PCR) used in this study were the same as described by Chen et al. (2017) and Song and Cui (2017). The primer pairs ITS5 and ITS4 for ITS regions, fRPB2-f5F and bRPB2-7.1R for the RPB2 gene, and EF1-983 F and EF1-1567R for the TEF gene used in this study are the same as in previous studies (White et al., 1990; Rehner, 2001; Matheny, 2005).

The PCR cycling schedules for different DNA sequences of ITS, RPB2, and TEF genes used in this study followed those used in Zhu et al. (2019) and Sun et al. (2020) with some modifications. The PCR procedure for ITS was the initial denaturation at 95°C for 3 min, followed by 35 cycles of denaturation at 94°C for 40 s, annealing at 54°C for 45 s, and extension at 72°C for 1 min, and a final extension at 72°C for 10 min. The PCR procedure for RPB2 was the initial denaturation at 94°C for 2 min, followed by 37 cycles of denaturation at 94°C for 45 s, annealing at 56°C for 90 s, and extension at 72°C for 2 min, and a final extension at 72°C for 10 min. The PCR procedure for TEF was the initial denaturation at 95°C for 3 min, followed by 35 cycles of denaturation at 94°C for 40 s, annealing at 54–57°C for 45 s and extension at 72°C for 1 min, and a final extension at 72°C for 10 min. The PCR products were purified and sequenced at the Beijing Genomics Institute (BGI), China, with the same primers. All newly generated sequences were deposited in GenBank (Table 1). Additional sequences for phylogenetic analyses were downloaded from GenBank (Table 1). All sequences were aligned in MAFFT 7 (Katoh and Standley, 2013)^1^ and manually adjusted in BioEdit (Hall, 1999). Alignments were spliced in Mesquite (Maddison and Maddison, 2017). The missing sequences were coded as “N,” ambiguous nucleotides were coded as “N” followed Chen et al. (2017). The final concatenated sequence alignment was deposited in TreeBase^2^ (submission ID: 27439).

**TABLE 1:** A list of species, specimens, and GenBank accession numbers of sequences used in this study.

Species name	Sample no.	Locality	GenBank accessions		
			ITS	RPB2	TEF
*Antrodia tanakae*	Cui 9743	China	KR605814	KR610833	KR610743
*A. tanakae*	Yuan 1106	China	KP715313	KR610835	KP715343
*Daedalea quercina*	Dai 12152	Czechia	KP171207	KR610809	KR610717
*D. quercina*	Dai 2260	Sweden	KR605792	KR610808	KR610718
*Fomitopsis abieticola*	Cui 10532	China	**MN148230**	**MN158174**	**MN161745**
*F. abieticola*	Cui 10521	China	**MN148231**	**—**	**MN161746**
*F. betulina*	Cui 10756	China	KR605797	KR610815	KR610725
*F. betulina*	Dai 11449	China	KR605798	KR610816	KR610726
*F. cana*	Cui 6239	China	JX435777	KR610761	KR610661
*F. cana*	Dai 9611	China	JX435776	KR610762	KR610660
*F. durescens*	Overholts 4215	United States	KF937293	—	—
*F. durescens*	O 10796	Venezuela	KF937292	KR610766	KR610669
*F. hengduanensis*	Cui 16259	China	**MN148232**	**MN158175**	**MN161747**
*F. hengduanensis*	Cui 17056	China	**MN148233**	**MN158176**	**MN161748**
*F. kesiyae*	Cui 16437	Vietnam	**MN148234**	**MN158177**	**MN161749**
*F. kesiyae*	Cui 16466	Vietnam	**MN148235**	**MN158178**	**MN161750**
*F. massoniana*	Cui 2848	China	**MN148236**	**—**	**MN161751**
*F. massoniana*	Cui 9058	China	**MN148237**	**—**	**MN161752**
*F. massoniana*	Cui 11288	China	**MN148238**	**MN158179**	**MN161753**
*F. massoniana*	Cui 11304	China	**MN148239**	**—**	**MN161754**
*F. meliae*	Dai 10035	China	KR605774	—	KR610683
*F. meliae*	Ryvarden 16893	Unknown	KR605776	KR610775	KR610681
*F. mounceae*	AFTOL ID 770	United States	AY786056	AY864874	AY705967
*F. mounceae*	DR 366	United States	KF169624	KF169693	KF178349
*F. mounceae*	JAG 08 19	United States	KF169626	KF169695	KF178351
*F. mounceae*	JEH 78	Canada	KF169629	KF169698	KF178354
*F. mounceae*	Tuomo Niemelä 2530	Canada	**MN148240**	**—**	**MN161755**
*F. mounceae*	Teuvo Ahti 60351	Canada	**MN148241**	**—**	**MN161756**
*F. mounceae*	OM 18782	United States	**MN148242**	**—**	**MN161757**
*F. mounceae*	Spirin 8367	United States	**MN148243**	**—**	**MN161758**
*F. ochracea*	HHB 19692	United States	KF169594	KF169663	KF178319
*F. ochracea*	HHB 19670	United States	KF169593	KF169662	KF178318
*F. ochracea*	JEH 38	United States	KF169603	KF169672	KF178328
*F. ochracea*	LT 18	United States	KF169616	KF169685	KF178341
*F. ochracea*	OM 18568	United States	**MN148244**	**—**	**MN161759**
*F. ochracea*	OM 18673	United States	**MN148245**	**—**	**MN161760**
*F. ochracea*	Spirin 8165	United States	**MN148246**	**—**	**MN161761**
*F. pinicola*	FCUG 2056	Sweden	KF169654	KF169723	KF178379
*F. pinicola*	HK 19330	Russia	KF169655	KF169724	KF178380
*F. pinicola*	LT 323	Estonia	KF169651	KF169720	KF178376
*F. pinicola*	LT 319	Estonia	KF169652	KF169721	KF178377
*F. pinicola*	AT Fp 1	Sweden	MK208852	MK236362	MK236359
*F. pinicola*	AT Fp 2	Sweden	MK208853	MK236363	MK236360
*F. schrenkii*	FP 105881 R	United States	KF169641	KF169710	KF178366
*F. schrenkii*	JEH 144	United States	KF169621	MK208857	MK236355
*F. schrenkii*	JEH 150	United States	KF169622	MK208858	MK236356
*F. schrenkii*	JV 1209/61 J	United States	**MN148247**	**MN158180**	**MN161762**
*F. schrenkii*	Inkeri Ahonen 58	United States	**MN148248**	**—**	**MN161763**
*F. subpinicola*	Cui 9836	China	**MN148249**	**MN158181**	**MN161764**
*F. subpinicola*	Cui 9819	China	**MN148250**	**—**	**MN161765**
*F. subpinicola*	Dai 11101	China	**MN148251**	**MN158182**	**MN161766**
*F. subpinicola*	Dai 11206	China	**MN148252**	**MN158183**	**MN161767**
*F. subpinicola*	Dai 13480	China	**MN148253**	**MN158184**	**MN161768**
*F. subpinicola*	Yuan 4912	China	**MN148254**	**—**	**MN161769**
*F. subtropica*	Cui 10578	China	KR605787	KR610791	KR610698
*F. subtropica*	Cui 10140	China	JQ067651	KR610789	KR610699
*F. tianshanensis*	Wei 1568	China	**MN148255**	**—**	**MN161770**
*F. tianshanensis*	Wei 1473a	China	**MN148256**	**—**	**MN161771**
*F. tianshanensis*	Wei 1462a	China	**MN148257**	**—**	**MN161772**
*F. tianshanensis*	Cui 16821	China	**MN148258**	**—**	**MN161773**
*F. tianshanensis*	Cui 16823	China	**MN148259**	**—**	**MN161774**
*F. tianshanensis*	Cui 16825	China	**MN148260**	**—**	**MN161775**
*F. tianshanensis*	Cui 16828	China	**MN148261**	**—**	**MN161776**
*F. tianshanensis*	Cui 16830	China	**MN148262**	**—**	**MN161777**
*Laetiporus zonatus*	Dai 13633	China	KX354481	KX354676	KX354635
*L. zonatus*	Cui 10404	China	KF951283	KT894797	KX354639
*Niveoporofomes spraguei*	JV 0509/62	United States	KR605786	KR610788	KR610697
*N. spraguei*	4638	France	KR605784	KR610786	KR610696
*Rhodofomes roseus*	Cui 10520	China	KC507162	KR610783	KR610692
*R. roseus*	Cui 10633	China	KR605782	KR610784	KR610693
*Rhodofomitopsis feei*	LR 14115	Costa Rica	KF999923	—	—
*R. feei*	JV 0610/K9-Kout	Mexico	KF999922	—	KR610673
*Rubellofomes cystidiatus*	Cui 5481	China	KF937288	KR610765	KR610667
*R. cystidiatus*	Yuan 6304	China	KR605769	—	KR610668

*New sequences are shown in bold.*

Phylogenetic analyses approaches used in this study followed Han et al. (2016) and Cui et al. (2019). The congruences of the 3-gene (ITS, RPB2 and TEF) were evaluated with the incongruence length difference (ILD) test (Farris et al., 1994) implemented in PAUP^∗^ 4.0b10 (Swofford, 2002), under heuristic search and 1000 homogeneity replicates. The sequences of *Daedalea quercina* (L.) Pers obtained from GenBank were used as outgroups for the phylogeny of the *Fomitopsis pinicola* complex, and sequences of *Laetiporus zonatus* B.K. Cui & J. Song were used as outgroups for the phylogeny of the *Fomitopsis pinicola* complex and related taxa. A maximum parsimony (MP) analysis was performed in PAUP^∗^ version 4.0b10 (Swofford, 2002). A maximum likelihood (ML) analysis was performed in RAxmL v.7.2.8 with a GTR + G + I model (Stamatakis, 2006). Bayesian inference (BI) was calculated by MrBayes 3.1.2 (Ronquist and Huelsenbeck, 2003) with a general time reversible (GTR) model of DNA substitution and a gamma distribution rate variation across sites determined by MrModeltest 2.3 (Posada and Crandall, 1998; Nylander, 2004). Clade robustness was assessed using a bootstrap (BT) analysis with 1000 replicates (Felsenstein, 1985). The branch support was evaluated with a bootstrapping method of 1000 replicates (Hillis and Bull, 1993). Branches that received bootstrap supports for the MP and ML, greater than or equal to 75% and Bayesian posterior probabilities (BPP) greater than or equal to 0.95, were considered as significantly supported (Shen et al., 2019; Sun et al., 2020). The phylogenetic tree was visualized using FigTree v1.4.2^3^.

## Results

### Molecular Phylogeny

**FIGURE 1:**
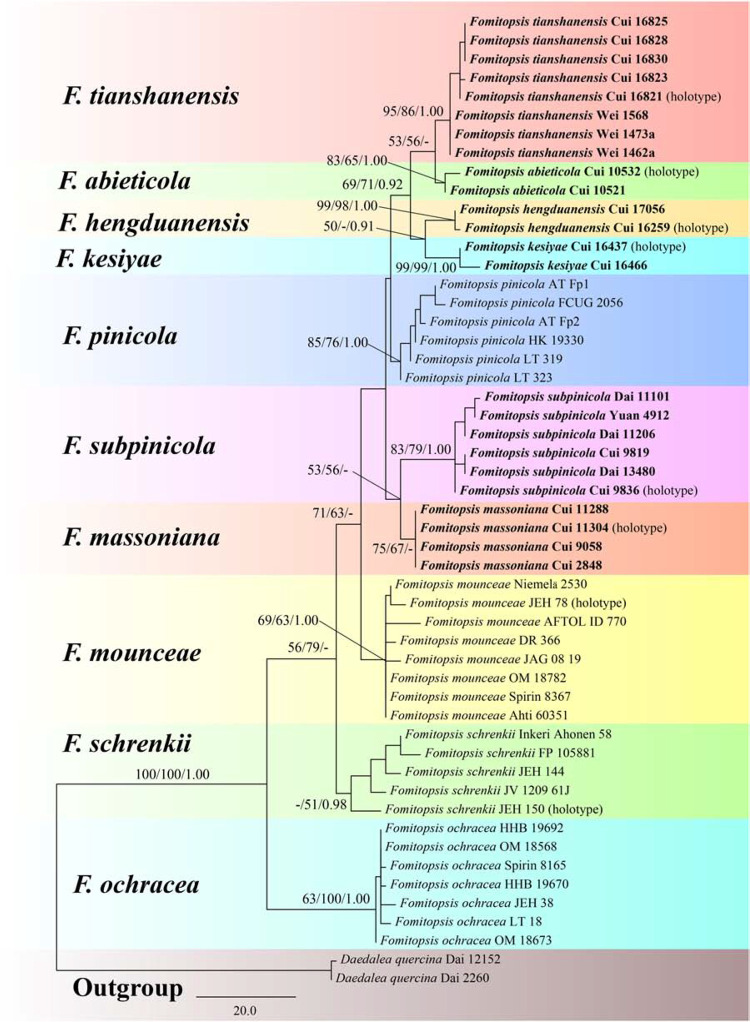
Maximum parsimony tree illustrating the phylogeny of the *Fomitopsis pinicola* complex based on the combined sequences dataset of ITS + RPB2 + TEF. *Daedalea quercina* served as the outgroup. Branches are labeled with maximum likelihood bootstrap higher than 50%, maximum parsimony bootstrap proportions higher than 50% and Bayesian posterior probabilities more than 0.95. Bold names = New species.

The combined 3-gene (ITS, RPB2, TEF) dataset to infer the phylogeny of species in the *Fomitopsis pinicola* complex included sequences from 52 fungal samples representing 11 taxa. The dataset had an aligned length of 1750 characters including gaps (553 characters for ITS, 641 characters for RPB2, 556 characters for TEF), of which 1403 characters were constant, 32 were variable and parsimony-uninformative, and 315 were parsimony-informative. Maximum parsimony analysis yielded 12 equally parsimonious trees (TL = 470, CI = 0.785, RI = 0.872, RC = 0.684, HI = 0.215). The best model for the combined ITS + RPB2 + TEF sequences dataset estimated and applied in the Bayesian analysis was GTR + I + G with equal frequency of nucleotides. Bayesian analysis and ML analysis resulted in a similar topology as the MP analysis, and only the MP tree inferred from the combined three-gene dataset is shown in Figure 1.

**FIGURE 2:**
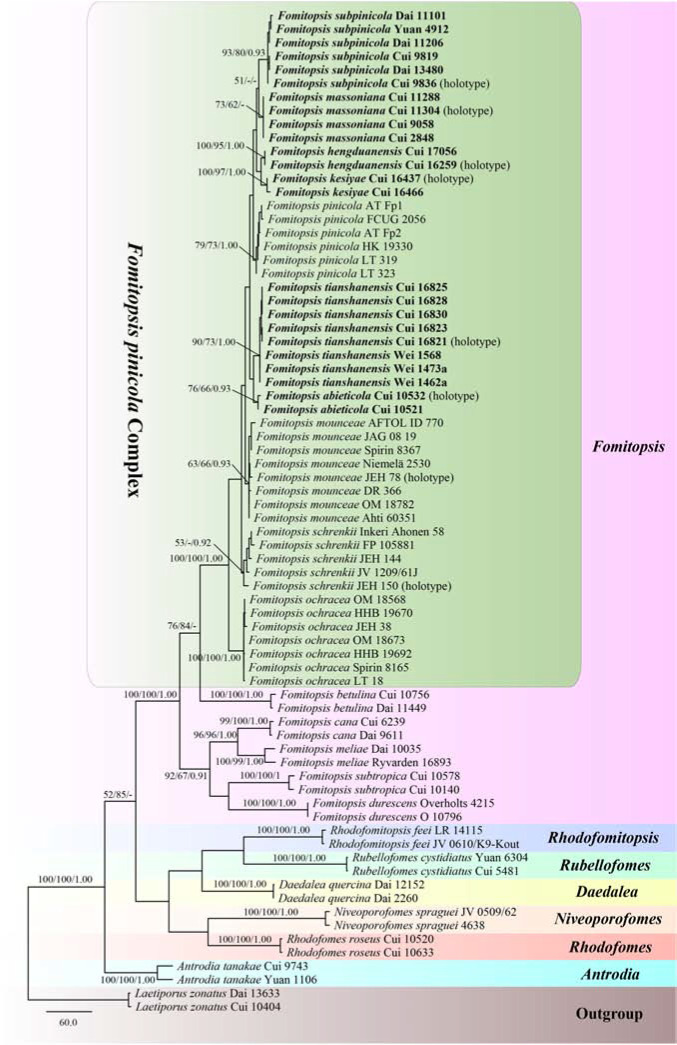
Maximum parsimony tree illustrating the phylogeny of the *Fomitopsis pinicola* complex and related group based on the combined sequences dataset of ITS + RPB2 + TEF. *Laetiporus zonatus* served as the outgroup. Branches are labeled with maximum likelihood bootstrap higher than 50%, maximum parsimony bootstrap proportions higher than 50% and Bayesian posterior probabilities more than 0.95. Bold names = New species.

The combined three-gene (ITS, RPB2, TEF) dataset infer the phylogeny of species in the *Fomitopsis pinicola* complex and the related group included sequences from 74 fungal samples representing 21 taxa. The dataset had an aligned length of 1848 characters including gaps (641 characters for ITS, 641 characters for RPB2, 566 characters for TEF), of which 1063 characters were constant, 41 were variable and parsimony-uninformative, and 744 were parsimony-informative. MP analysis yielded 10 equally parsimonious trees (TL = 2428, CI = 0.521, RI = 0.762, RC = 0.397, HI = 0.479). The best model for the concatenate sequence dataset estimated and applied in the Bayesian inference was GTR + I + G with an equal frequency of nucleotides. Bayesian analysis and ML analysis resulted in a similar topology as the MP analysis, and only the MP tree inferred from the combined three-gene sequences dataset is shown in Figure 2.

The phylogenetic trees (Figures 1, 2) generated by Maximum parsimony, Maximum likelihood and Bayesian analyses showed that the six new species, *Fomitopsis abieticola*, *F. hengduanensis*, *F. kesiyae*, *F. massoniana*, *F. subpinicola*, and *F. tianshanensis* grouped together with *F. mounceae*, *F. ochracea*, *F. pinicola*, and *F. schrenkii*, thus, the species number of the *F. pinicola* complex increased to 10 around the world.

### Taxonomy

**FIGURE 3:**
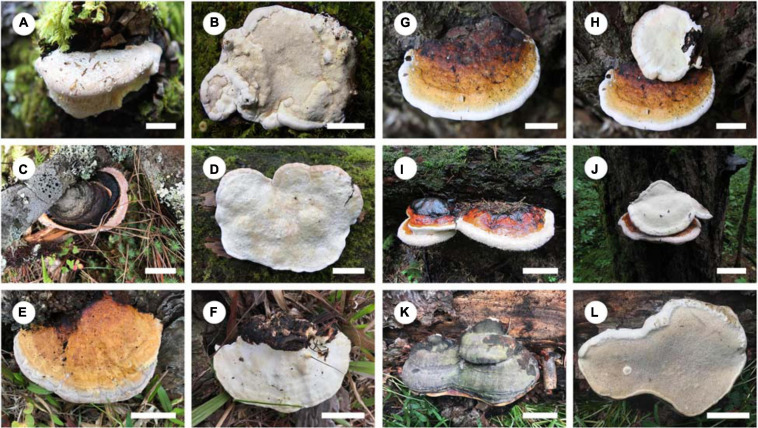
Basidiocarps of the *Fomitopsis pinicola* complex species. **(A,B)** *F*. *abieticola*; **(C,D)** *F*. *hengduanensis*; **(E,F)** *F*. *kesiyae*; **(G,H)** *F*. *massoniana*; **(I,J)** *F*. *subpinicola*; **(K,L)** *F*. *tianshanensis*. Bars: **A,B,D,E,F** = 2 cm; **G,H** = 1 cm; **C,I,J** = 3 cm; **K,L** = 5 cm.

**FIGURE 4:**
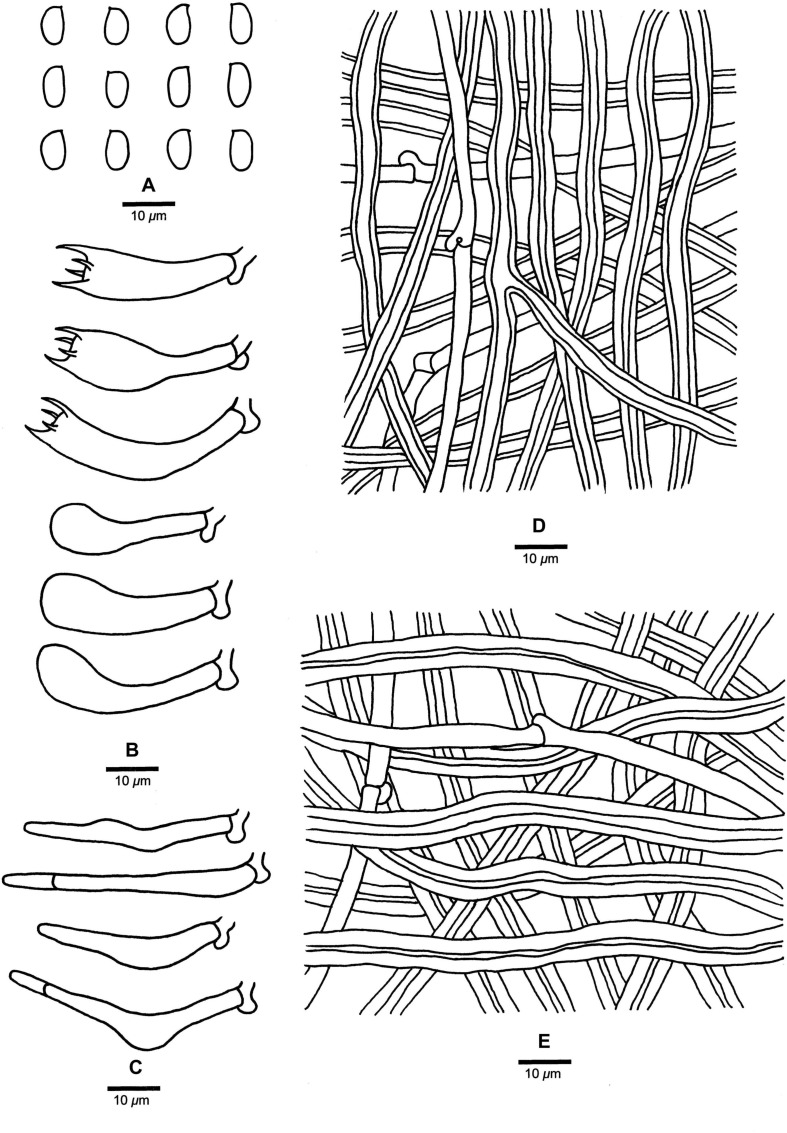
Microscopic structures of *Fomitopsis abieticola* (drawn from the holotype). **(A)** Basidiospores; **(B)** Basidia and basidioles; **(C)** Cystidioles; **(D)** Hyphae from trama; **(E)** Hyphae from context. Bars: **A–E** = 10 μm.

***Fomitopsis abieticola*** B.K. Cui, M.L. Han & Shun Liu, **sp. nov.** (Figures 3A,B, 4).

MycoBank: MB 838908

***Fomitopsis abieticola*** is characterized by its large pores (2–4 per mm), long cystidioles (17.5–50.2 × 4.3–9.5 μm), long basidia (20.8–40.5 × 5.5–11.5 μm) and big basidiospores (7–9 × 4–5 μm), and grows on *Abies*.

Type. — **CHINA**. Yunnan Province, Shangri-La County, Pudacuo National Park, on stump of *Abies*, 24 September 2011, *Cui 10532* (Holotype, BJFC 011427).

Etymology. —*Abieticola* (Lat.), refers to the host tree genus *Abies*.

Basidiocarps. —Annual to perennial, pileate, sessile, solitary, hard corky, without odor or taste when fresh, woody hard and light in weight upon drying. Pilei semicircular to ungulate, projecting up to 6.5 cm long, 8.5 cm wide, 2.5 cm thick at base. Pileal surface cream to pinkish buff when fresh, becoming honey-yellow to grayish brown when dry, glabrous, small nodules appear near the base, rough, azonate; margin cream, slightly paler than pileal surface, obtuse. Pore surface cream to pinkish buff when fresh, becoming buff to curry-yellow when dry; sterile margin distinct, white to cream when fresh, becoming olivaceous buff to clay-buff when dry, up to 10 mm wide; pores round to angular, 2–4 per mm; dissepiments slightly thick to thick, entire. Context cream to straw-yellow, woody hard, up to 1.5 cm thick. Tubes concolorous with pore surface, woody hard, up to 1 cm long.

Hyphal structure. —Hyphal system dimitic; generative hyphae bearing clamp connections; skeletal hyphae IKI–, CB–; tissues unchanged in KOH.

Context. —Generative hyphae infrequent, hyaline, thin-walled, rarely branched, 2.5–5 μm in diam; skeletal hyphae dominant, yellowish brown to cinnamon brown, thick-walled with a narrow lumen to subsolid, rarely branched, straight, interwoven, 2.3–8.2 μm in diam.

Tubes. —Generative hyphae infrequent, hyaline, thin-walled, rarely branched, 1.9–3.2 μm in diam; skeletal hyphae dominant, hyaline, thick-walled with a wide lumen, occasionally branched, more or less straight, interwoven, 2.2–7.2 μm in diam. Cystidia absent, but fusoid cystidioles occasionally present, hyaline, thin-walled, 17.5–50.2 × 4.3–9.5 μm. Basidia clavate, bearing four sterigmata and a basal clamp connection, 20.8–40.5 × 5.5–11.5 μm; basidioles dominant, in shape similar to basidia, but smaller.

Spores. —Basidiospores oblong-ellipsoid to ellipsoid, hyaline, thin-walled, smooth, IKI–, CB–, 7–9(–9.2) × (3.2–)4–5 μm, L = 7.85 μm, W = 4.26 μm, Q = 1.83–1.89 (*n* = 60/2).

Type of rot. —Brown rot.

Additional specimen (paratype) examined: **CHINA**. Yunnan Province, Shangri-La County, Pudacuo National Park, on stump of *Abies*, 24 September 2011, *Cui 10521* (BJFC 011416).

**FIGURE 5:**
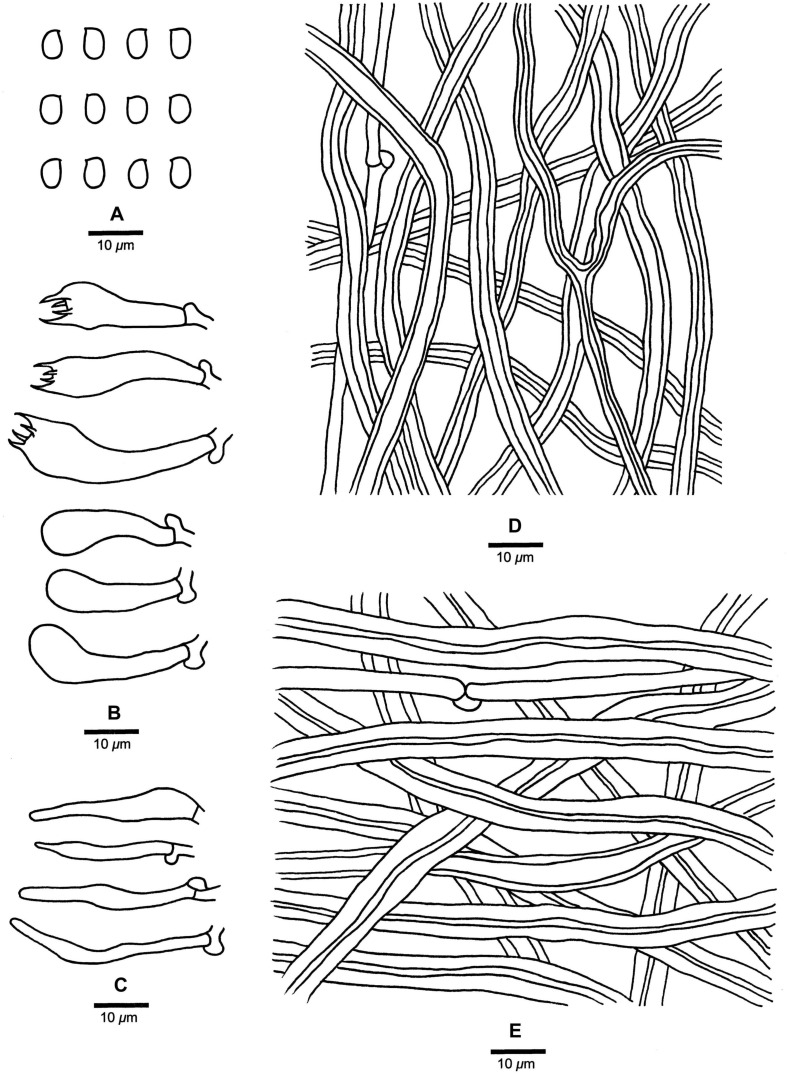
Microscopic structures of *Fomitopsis hengduanensis* (drawn from the holotype). **(A)** Basidiospores; **(B)** Basidia and basidioles; **(C)** Cystidioles; **(D)** Hyphae from trama; **(E)** Hyphae from context. Bars: **A–E** = 10 μm.

***Fomitopsis hengduanensis*** B.K. Cui & Shun Liu, **sp. nov.** (Figures 3C,D, 5).

MycoBank: MB 838909

***Fomitopsis hengduanensis*** is characterized by laccate pileus with pale dark gray to reddish brown surface at base and cream to flesh-pink toward the margin when fresh, oblong-ellipsoid to ellipsoid basidiospores (5.2–6 × 3.2–3.6 μm) and is distributed in high altitude areas of the Hengduan Mountains.

Type. —**CHINA**, Yunnan Province, Lanping County, Tongdian, Laojunshan of Hengduan Mountains, Luoguqing, on dead tree of *Picea*, 18 September 2017, *Cui 16259* (Holotype, BJFC 029558).

Etymology. —*Hengduanensis* (Lat.), refers to the species distributed in the area of Hengduan Mountains.

Basidiocarps. —Annual to perennial, pileate, sessile, solitary, hard corky, without odor or taste when fresh, woody hard and light in weight upon drying. Pilei applanate, semicircular to ungulate, projecting up to 7.5 cm long, 9 cm wide, 3 cm thick at base. Pileal surface laccate, colors varied but usually pale dark gray to reddish brown at base and cream to flesh-pink toward the margin when fresh, curry-yellow, mouse-gray to reddish brown at base and buff to clay-buff toward the margin when dry, glabrous, sulcate, concentrically zonate; margin acute to obtuse. Pore surface white to cream when fresh, becoming buff to straw-yellow when dry; sterile margin distinct, cream, up to 4 mm wide; pores round to angular, 6–8 per mm; dissepiments thick, entire. Context cream to straw-yellow, woody hard, up to 1.4 cm thick. Tubes concolorous with pore surface, woody hard, up to 0.5 cm long.

Hyphal structure. —Hyphal system dimitic; generative hyphae bearing clamp connections; skeletal hyphae IKI–, CB–; tissues unchanged in KOH.

Context. —Generative hyphae infrequent, hyaline, thin- to slightly thick-walled, rarely branched, 1.9–4.3 μm in diam; skeletal hyphae dominant, hyaline to pale yellowish, thick-walled with a narrow lumen to subsolid, rarely branched, straight, interwoven, 2–8.5 μm in diam.

Tubes. —Generative hyphae infrequent, hyaline, thin-walled, rarely branched, 1.3–3.5 μm in diam; skeletal hyphae dominant, hyaline, thick-walled with a wide lumen, occasionally branched, more or less straight, interwoven, 1.7–7.5 μm in diam. Cystidia absent, but fusoid cystidioles occasionally present, hyaline, thin-walled, 13.2–36.5 × 2.5–5.4 μm. Basidia clavate, bearing four sterigmata and a basal clamp connection, 16.6–34.5 × 5.4–10.2 μm; basidioles dominant, in shape similar to basidia, but smaller.

Spores. —Basidiospores oblong-ellipsoid to ellipsoid, hyaline, thin-walled, smooth, IKI–, CB–, (5–)5.2–6(–6.2) × (3–)3.2–3.6(–4) μm, L = 5.44 μm, W = 3.41 μm, Q = 1.57–1.63 (*n* = 60/2).

Type of rot. —Brown rot.

Additional specimen (paratype) examined: **CHINA**. Yunnan Province, Lijiang, Yulong xueshan of Hengduan Mountains, on fallen trunk of *Picea*, 16 September 2018, *Cui 17056* (BJFC 030355).

**FIGURE 6:**
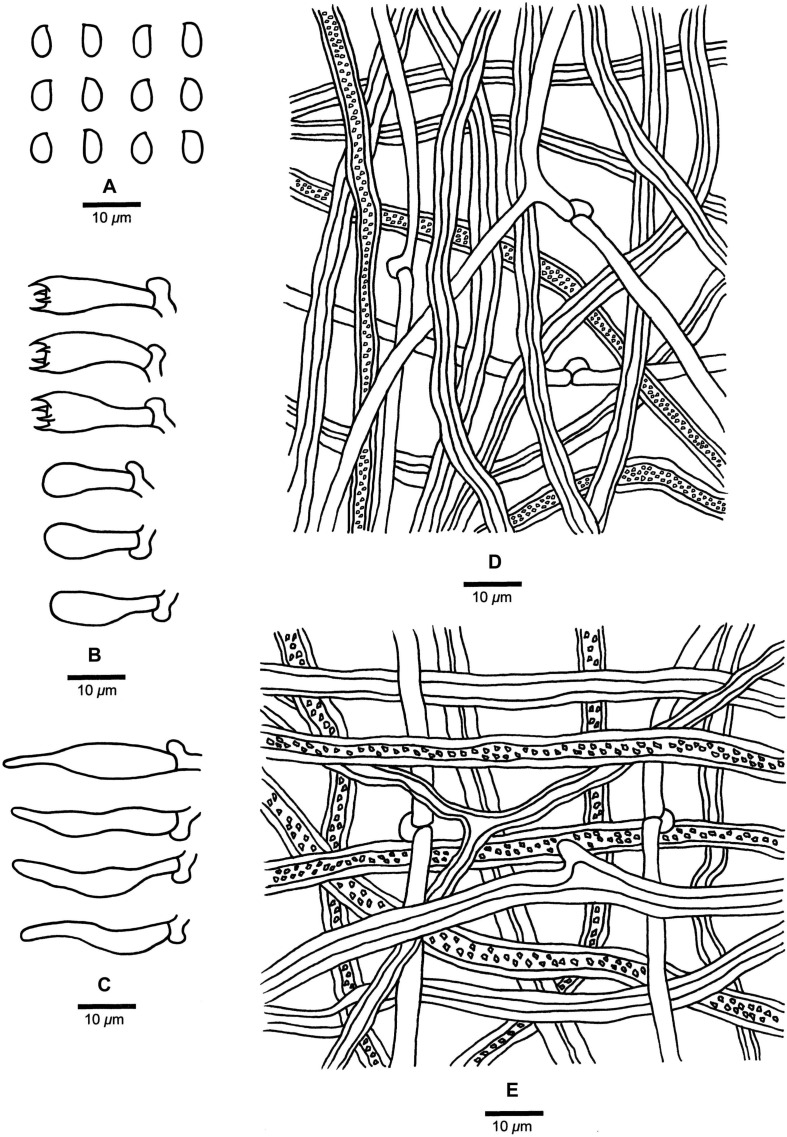
Microscopic structures of *Fomitopsis kesiyae* (drawn from the holotype). **(A)** Basidiospores; **(B)** Basidia and basidioles; **(C)** Cystidioles; **(D)** Hyphae from trama; **(E)** Hyphae from context. Bars: **A–E** = 10 μm.

***Fomitopsis kesiyae*** B.K. Cui & Shun Liu, **sp. nov.** (Figures 3E,F, 6).

MycoBank: MB 838910

***Fomitopsis kesiyae*** is characterized by its buff yellow to orange-yellow buff pileal surface when fresh, reddish brown to yellowish brown when dry, and grows on *Pinus kesiya* and is distributed in tropical areas of Vietnam.

*Type*. — **VIETNAM**. Dam Dong Province, Da Lat, Bidoup Nui Ba National Park, on living tree of *Pinus kesiya*, 15 October 2017, *Cui 16437* (Holotype, BJFC 029736).

*Etymology*. — *Kesiyae* (Lat.), refers to the host tree species *Pinus kesiya*.

*Basidiocarps*. — Annual, pileate, sessile, hard corky, without odor or taste when fresh, woody hard and light in weight upon drying. Pilei applanate, semicircular to sectorial, projecting up to 4.7 cm long, 6.5 cm wide, 4 cm thick at base. Pileal surface laccate, buff yellow to orange-yellow buff when fresh, becoming reddish brown to yellowish brown when dry, glabrous, sulcate, azonate; margin cream, distinctly paler than the pileal surface, obtuse. Pore surface white to cream when fresh, olivaceous buff to cinnamon-buff when dry; sterile margin distinct, buff to honey-yellow, up to 3 mm wide; pores round to angular, 6–8 per mm, dissepiments thick, entire. Context cream to straw-yellow, corky, up to 1.2 cm thick. Tubes concolorous with pore surface, hard corky, up to 1 cm long.

*Hyphal structure*. — Hyphal system dimitic; generative hyphae bearing clamp connections; skeletal hyphae IKI–, CB–; tissues unchanged in KOH. Small polyhedric or irregular crystals present among context and tubes.

*Context*. — Generative hyphae infrequent, hyaline, thin- to slightly thick-walled, rarely branched, 1.9–4.2 μm in diam; skeletal hyphae dominant, yellowish brown to cinnamon brown, thick-walled with a wide to narrow lumen, occasionally branched, straight to flexuous, interwoven, 2.2–9.2 μm in diam.

*Tubes*. — Generative hyphae infrequent, hyaline, thin-walled, occasionally branched, 1.9–3 μm in diam; skeletal hyphae dominant, hyaline to pale yellowish, thick-walled with a wide to narrow lumen, rarely branched, more or less straight, interwoven, 1.7–6.2 μm in diam. Cystidia absent, but fusoid cystidioles occasionally present, hyaline, thin-walled, 11.5–30.4 × 2.6–6 μm. Basidia clavate, bearing four sterigmata and a basal clamp connection, 16–20.3 × 4.8–7.2 μm; basidioles dominant, in shape similar to basidia, but smaller.

*Spores*. — Basidiospores oblong-ellipsoid to ellipsoid, hyaline, thin-walled, smooth, IKI–, CB–, (4.5–)4.8–5.3(–6) × (2.8–)3–3.5(–4) μm, L = 5.04 μm, W = 3.11 μm, Q = 1.60–1.65 (*n* = 60/2).

*Type of rot*. — Brown rot.

Additional specimen (paratype) examined: **VIETNAM**. Dam Dong Province, Da Lat, Bidoup Nui Ba National Park, on fallen trunk of *Pinus kesiya*, 16 October 2017, *Cui 16466* (BJFC 029765).

**FIGURE 7:**
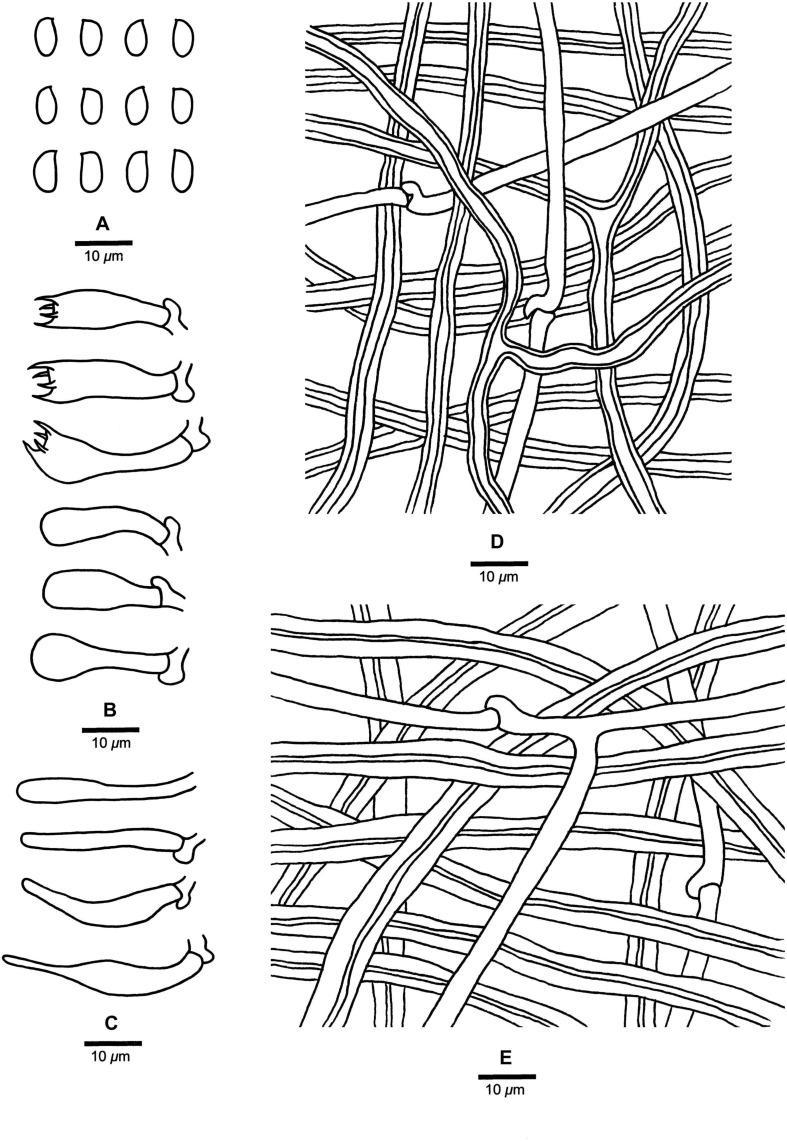
Microscopic structures of *Fomitopsis massoniana* (drawn from the holotype). **(A)** Basidiospores; **(B)** Basidia and basidioles; **(C)** Cystidioles; **(D)** Hyphae from trama; **(E)** Hyphae from context. Bars: **A–E** = 10 μm.

***Fomitopsis massoniana*** B.K. Cui, M.L. Han & Shun Liu, **sp. nov.** (Figures 3G,H, 7).

MycoBank: MB 838911

***Fomitopsis massoniana*** is characterized by its effused-reflexed to pileate basidiocarps, applanate to triquetrous or irregular pilei with buff-yellow to apricot-orange pileal surface when fresh, buff to grayish brown when dry, a white to cream pore surface when fresh, cream to buff when dry, and grows on *Pinus massoniana*.

*Type*. — **CHINA**. Fujian Province, Wuping County, Liangyeshan Nature Reserve, on dead tree of *Pinus massoniana*, 25 October 2013, *Cui 11304* (Holotype, BJFC 015420).

*Etymology*. — *Massoniana* (Lat.), refers to the host tree species *Pinus massoniana*.

*Basidiocarps*. — Annual, effused-reflexed to pileate, sessile, hard corky, without odor or taste when fresh, woody hard and light in weight upon drying. Pilei applanate to triquetrous or irregular, projecting up to 4 cm long, 4.2 cm wide, 1.5 cm thick at base. Pileal surface laccate, buff-yellow to apricot-orange when fresh, becoming buff to grayish brown when dry, glabrous, sulcate, azonate; margin white to cream, obtuse. Pore surface white to cream when fresh, turning cream to buff when dry; sterile margin distinct, cream, up to 4 mm wide; pores round, 5–7 per mm, dissepiments thick, entire. Context cream to straw-yellow, woody hard, up to 0.8 cm thick. Tubes concolorous with pore surface, woody hard, up to 0.4 cm long.

*Hyphal structure*. — Hyphal system dimitic; generative hyphae bearing clamp connections; skeletal hyphae IKI–, CB–; tissues unchanged in KOH.

*Context*. — Generative hyphae infrequent, hyaline, thin- to slightly thick-walled, occasionally branched, 2–4.5 μm in diam; skeletal hyphae dominant, hyaline, thick-walled with a narrow lumen to subsolid, rarely branched, straight, interwoven, 2.2–8.2 μm in diam.

*Tubes*. — Generative hyphae infrequent, hyaline, thin-walled, rarely branched, 1.8–4 μm in diam; skeletal hyphae dominant, hyaline, thick-walled with a narrow lumen to narrow lumen, occasionally branched, more or less straight, interwoven, 2–7.2 μm in diam. Cystidia absent, but fusoid cystidioles occasionally present, hyaline, thin-walled, 14.8–36 × 3.8–6 μm. Basidia clavate, bearing four sterigmata and a basal clamp connection, 17–26.5 × 5.5–7.9 μm; basidioles dominant, in shape similar to basidia, but smaller.

*Spores*. — Basidiospores oblong-ellipsoid, hyaline, thin-walled, smooth, IKI–, CB–, (5.8–)6.2–7.3(–7.6) × (3–)3.3–4 μm, L = 6.91 μm, W = 3.53 μm, Q = 1.93–1.99 (*n* = 90/3).

*Type of rot*. — Brown rot.

Additional specimens (paratypes) examined: **CHINA**. Fujian Province, Wuping County, Liangyeshan Nature Reserve, on dead tree of *Pinus massoniana*, 25 October 2013, *Cui 11288* (BJFC 015404); Wuyishan County, Longchuan Valley, on dead tree of *Pinus massoniana*, 16 October 2005, *Cui 2848* (BJFC 000719); Guangdong Province, Fengkai County, Heishiding Nature Reserve, on fallen trunk of *Pinus massoniana*, 2 July 2010, *Cui 9058* (BJFC 007996).

**FIGURE 8:**
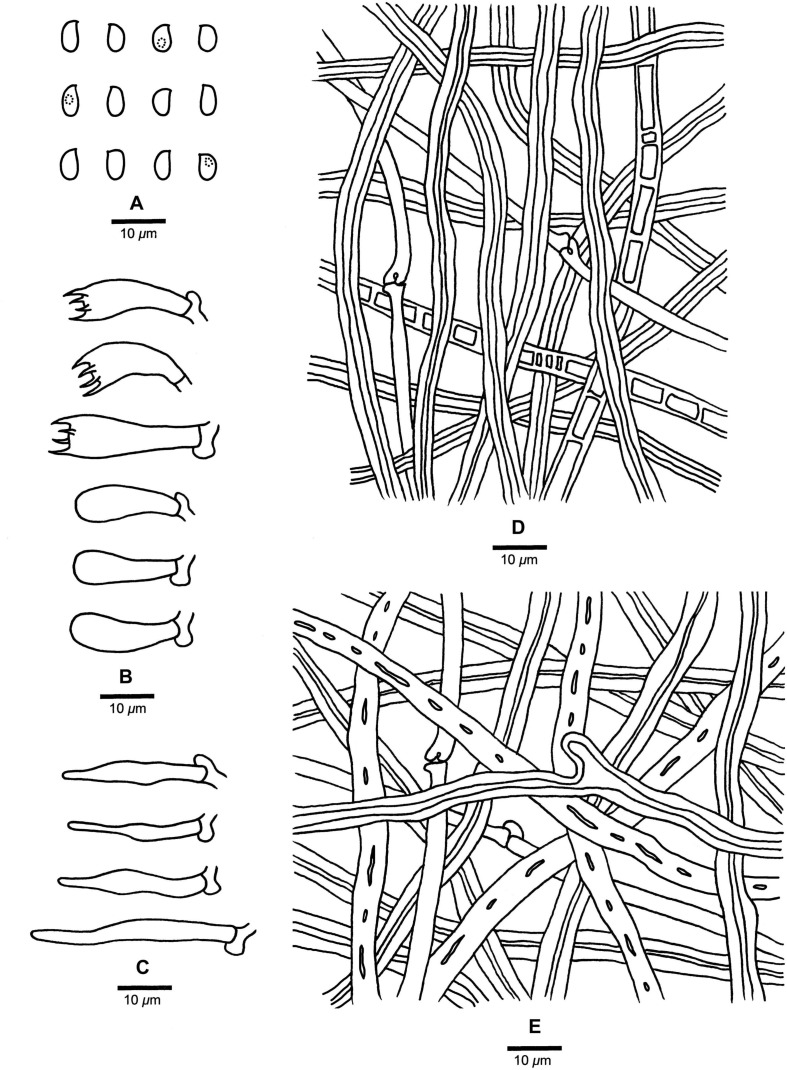
Microscopic structures of *Fomitopsis subpinicola* (drawn from the holotype). **(A)** Basidiospores; **(B)** Basidia and basidioles; **(C)** Cystidioles; **(D)** Hyphae from trama; **(E)** Hyphae from context. Bars: **A–E** = 10 μm.

***Fomitopsis subpinicola*** B.K. Cui, M.L. Han & Shun Liu, **sp. nov.** (Figures 3I,J, 8).

MycoBank: MB 838912

***Fomitopsis subpinicola*** is characterized by its apricot-orange, scarlet to fuscous pileal surface when fresh, reddish brown to dark brown when dry, occasionally septated skeletal hyphae and is distributed in Northeast China.

*Type*. — **CHINA**. Heilongjiang Province, Yichun, Fenglin Nature Reserve, on fallen trunk of *Pinus koraiensis*, 2 August 2011, *Cui 9836* (Holotype, BJFC 010729).

*Etymology*. — *Subpinicola* (Lat.), refers to the new species resembling *F. pinicola* in morphology.

*Basidiocarps*. — Annual, pileate, sessile, hard corky, without odor or taste when fresh, woody hard and light in weight upon drying. Pilei applanate, circular to sectorial, projecting up to 7.5 cm long, 8.5 cm wide, 4.5 cm thick at base. Pileal surface laccate, apricot-orange, scarlet to fuscous when fresh, becoming reddish brown to dark brown upon drying, glabrous, sulcate, azonate; margin white to cream, distinctly paler than the pileal surface, obtuse. Pore surface white to cream when fresh, turning buff yellow to buff when dry; sterile margin distinct, white to cream, up to 6 mm wide; pores round, 6–8 per mm, dissepiments thick, entire. Context cream to straw-yellow, woody hard, up to 1.2 cm thick. Tubes concolorous with pore surface, woody hard, up to 0.5 cm long.

*Hyphal structure*. — Hyphal system dimitic; generative hyphae bearing clamp connections; skeletal hyphae IKI–, CB–; tissues unchanged in KOH.

*Context*. — Generative hyphae infrequent, hyaline, thin- to slightly thick-walled, rarely branched, 2–3.2 μm in diam; skeletal hyphae dominant, yellowish brown to cinnamon brown, thick-walled with a narrow lumen to subsolid, occasionally branched, straight, interwoven, 2.2–6.8 μm in diam.

*Tubes*. — Generative hyphae infrequent, hyaline, thin-walled, rarely branched, 1.8–3 μm in diam; skeletal hyphae dominant, yellowish brown to cinnamon brown, thick-walled with a wide to narrow lumen, occasionally septate, without clamps, rarely branched, straight, interwoven, 1.9–6.2 μm in diam. Cystidia absent, but fusoid cystidioles occasionally present, hyaline, thin-walled, 14.5–34.6 × 3.2–7.2 μm. Basidia clavate, bearing four sterigmata and a basal clamp connection, 16–24.5 × 4.5–9 μm; basidioles dominant, in shape similar to basidia, but smaller.

*Spores*. — Basidiospores oblong-ellipsoid to ellipsoid, hyaline, thin-walled, smooth, IKI–, CB–, (4–)4.3–5.5(–5.9) × (2.5–)2.7–3.3(–3.5) μm, L = 4.94 μm, W = 2.97 μm, Q = 1.65–1.69 (*n* = 90/3).

*Type of rot*. — Brown rot.

Additional specimens (paratypes) examined: **CHINA**. Heilongjiang Province, Yichun, Fenglin Nature Reserve, on fallen trunk of *Pinus koraiensis*, 1 August 2011, *Cui 9819* (BJFC 010712); Tangyuan County, Daliangzihe Forest Park, on living tree of *Pinus koraiensis*, 26 August 2008, *Yuan 4912* (BJFC 015654); Inner Mongolia, Genhe, Greater Khingan Mountains Nature Reserve, on *Larix*, 28 August 2009, *Dai 11101* (BJFC 015660), *Dai 11206* (BJFC 015661); Jilin Province, Antu County, Changbaishan Nature Reserve, on fallen trunk of *Betula*, 7 September 2013, *Dai 13480* (BJFC 014941).

**FIGURE 9:**
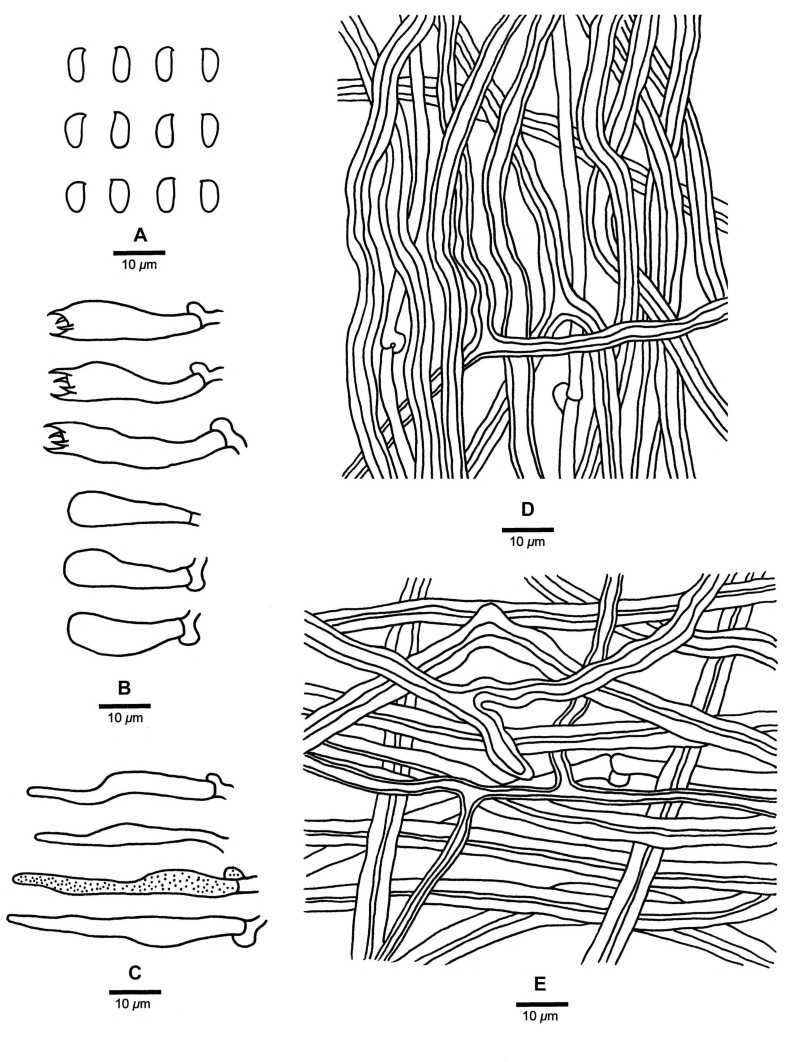
Microscopic structures of *Fomitopsis tianshanensis* (drawn from the holotype). **(A)** Basidiospores; **(B)** Basidia and basidioles; **(C)** Cystidioles; **(D)** Hyphae from trama; **(E)** Hyphae from context. Bars: **A–E** = 10 μm.

***Fomitopsis tianshanensis*** B.K. Cui & Shun Liu, **sp. nov.** (Figures 3K,L, 9).

MycoBank: MB 838913

***Fomitopsis tianshanensis*** is characterized by its effused-reflexed to pileate basidiocarps with soft corky texture when fresh, large pores (1–3 per mm) and long tubes (up to 2.5 cm), grows on *Picea* and is distributed in Tianshan Mountains Xinjiang, China.

*Type*. — **CHINA**. Xinjiang Autonomous Region, Fukang County, Tianshan Tianchi Nature Reserve, on fallen trunk of *Picea schrenkiana*, 4 July 2018, *Cui 16821* (Holotype, BJFC 030120).

*Etymology*. — *Tianshanensis* (Lat.), refers to the species located at the Tianshan regions.

*Basidiocarps*. — Annual to perennial, effused-reflexed to pileate, sessile, soft corky, without odor or taste when fresh, hard corky and light in weight upon drying. Pilei applanate, semicircular to ungulate, projecting up to 11 cm long, 20 cm wide, 7 cm thick at base. Pileal surface dark bluish gray to yellowish brown when fresh, becoming fawn to deep olive when dry, slightly velutinate, small nodules appear near the base, rough, azonate; margin cream to cinnamon, obtuse to acute. Pore surface cream to pinkish buff when fresh, becoming faint yellow to light pink when dry; sterile margin distinct, cream to buff, up to 3 mm wide; pores mostly round, occasionally angular, 1–3 per mm, dissepiments thick, entire. Context cream to buff, corky, up to 3.5 cm thick. Tubes concolorous with pore surface, hard corky, up to 2.5 cm long.

*Hyphal structure*. — Hyphal system dimitic; generative hyphae bearing clamp connections; skeletal hyphae IKI–, CB–; tissues unchanged in KOH.

*Context*. — Generative hyphae infrequent, hyaline, thin- to slightly thick-walled, rarely branched, 2–4 μm in diam; skeletal hyphae dominant, yellowish brown to cinnamon brown, thick-walled with a narrow lumen to subsolid, occasionally branched, straight to flexuous, interwoven, 2.2–7.2 μm in diam.

*Tubes*. — Generative hyphae infrequent, hyaline, thin-walled, rarely branched, 1.9–3.2 μm in diam; skeletal hyphae dominant, hyaline to pale yellowish, thick-walled with a wide lumen, occasionally branched, straight to flexuous, 2–6.9 μm in diam, interwoven. Cystidia absent, sometimes skeletal hyphae penetrated into the hymenium, but not forming typical catahymenium; cystidioles present, fusoid, hyaline, thin-walled, 15.5–44 × 3.3–6.5 μm. Basidia clavate, bearing four sterigmata and a basal clamp connection, 17–32.5 × 4.2–9.5 μm; basidioles dominant, in shape similar to basidia, but smaller.

*Spores*. — Basidiospores oblong-ellipsoid, sometimes tapering at apiculus, hyaline, thin-walled, smooth, IKI–, CB–, (6–)6.3–7(–7.2) × (3–)3.2–3.8(–4) μm, L = 6.62 μm, W = 3.52 μm, Q = 1.85–1.93 (*n* = 90/3).

*Type of rot*. — Brown rot.

Additional specimens (paratypes) examined: **CHINA**. Xinjiang Autonomous Region, Urumqi, Nanshan Park, on fallen trunk of *Picea schrenkiana*, 5 July 2018, *Cui 16823* (BJFC 030122), *Cui 16825* (BJFC 030124), *Cui 16828* (BJFC 030127); Shawan County, Lujiaowan Park, on stump of *Picea schrenkiana*, 6 July 2018, *Cui 16830* (BJFC 030129).

### Other Specimens of the *Fomitopsis pinicola* Complex Examined

*Fomitopsis mounceae*. **CANADA**. 3 August 2000, *Teuvo Ahti 60351* (H); on *Picea glauca*, 25 July 1982, *Tuomo Niemelä 2530* (H). **UNITED STATES**. On *Betula*, 15 September 2014, *OM 18782* (H); on *Tsuga heterophylla*, 11 September 2014, *Spirin 8367* (H).

*Fomitopsis ochracea*. **UNITED STATES**. *OM 18568* (H), *OM 18673* (H); on *Picea*, *Spirin 8165* (H).

*Fomitopsis schrenkii*. **UNITED STATES**. 7 September 1992, *Inkeri Ahonen 58* (BJFC 013921); Turkey Creek, Chricahua Mountain, Arizona, on *Douglas fir*, September 2012, *Josef Vlasák 1209/61-J* (BJFC 015604).

*Fomitopsis pinicola*. **FINLAND**. Helsinki, Vantaa, Tamisto Nature Reserve, on fallen trunk of *Picea*, 16 August 2012, *Dai 12869* (BJFC 013149), *Dai 12870* (BJFC 013150). **ITALY**. Roma, Trentino Altoadie, Trento, Molveno, on *Picea*, 28 April 2005, *Dai 6553* (IFP). **POLAND**. On dead tree of *Pinus*, 3 October 2014, *Dai 14841* (BJFC 017955); Lesser Poland Voivodeship, Gorce National Park, on *Picea abies*, 9 July 1985, *Pekka Nuorteva* (H).

## Discussion

Based on the phylogenetic analyses, 10 species of the *Fomitopsis pinicola* complex grouped together (Figures 1, 2), including six new species from East Asia: *F. abieticola*, *F. hengduanensis*, *F. kesiyae*, *F. massoniana*, *F. subpinicola*, and *F. tianshanensis*. The main morphological characters of species in the *F. pinicola* complex are provided in Table 2.

**TABLE 2:** A comparison of species in the *Fomitopsis pinicola* complex.

Species	Distribution	Basidio-	Pileal surface	Host	Pores (per mm)	Cystidia (μm)	Basidiospores		References
					
		carps	when fresh				L × W (μm)	Q = L/W	
*F. abieticola*	Southwestern China	Annual to perennial; pileate	Cream to pinkish buff	*Abies*	2–4	17.5–50.2 × 4.3–9.5	7–9 × 4–5	1.83–1.89	This study
*F. hengduanensis*	High altitude areas of the Hengduan Mountains of southwestern China	Annual to perennial; pileate	Pale dark gray to reddish brown at base and cream to flesh-pink toward the margin	*Picea*, *Pinus*	6–8	13.2–36.5 × 2.5–5.4	5.2–6 × 3.2–3.6	1.57–1.63	This study
*F. kesiyae*	Tropical areas of Vietnam	Annual; pileate	Buff yellow to orange-yellow buff	*Pinus kesiya*	6–8	11.5–30.4 × 2.6–6	4.8–5.3 × 3–3.5	1.60–1.65	This study
*F. massoniana*	Southeastern China	Annual; effused-reflexed to pileate	Buff-yellow to apricot-orange	*Pinus massoniana*	5–7	14.8–36 × 3.8–6	6.2–7.3 × 3.3–4	1.85–1.9	This study
*F. mounceae*	Canada, northern United States	Perennial; pileate	Brownish orange to black at base and pale orange to grayish orange toward the margin	*Abies*, *Betula*, *Larix*, *Picea*, *Populus*, *Pseudotsuga*	3–5	16–35 × 3–6.5	5.8–6.6 × 3.4–4	1.7–1.9	Haight et al., 2019
*F. ochracea*	Canada, northern United States	Perennial; pileate	Brownish gray to grayish brown at base and orange White to pale orange toward the margin	*Abies*, *Picea*, *Populus*	4–5	20–40 × 4–6.5	5.1–5.9 × 3.6–4	1.3–1.4	Haight et al., 2019
*F. pinicola*	Europe	Perennial; pileate	Brownish orange to black at base and buff-yellow to cinnamon toward the margin	Mostly on *Picea* and *Pinus*, occasionally on other different gymnosperm or angiosperm wood	4–6	18–60 × 3–9	6–9 × 3–4.5	1.8–2.2	Ryvarden and Melo, 2014; Haight et al., 2016, 2019
*F. schrenkii*	Western and southwestern United States	Perennial; effused-reflexed to pileate	Grayish orange to olive brown at base and grayish orange to grayish yellow toward the margin	*Abies*, *Picea*, *Pinus*, *Pseudotsuga*	3–4	16–30 × 3–8	5.7–6.7 × 3.7–4.2	1.5–1.6	Haight et al., 2019
*F. subpinicola*	Northeastern China	Annual; pileate	Apricot-orange, scarlet to fuscous	Mostly on *Pinus koraiensis*, occasionally on *Larix*, *Betula*	6–8	14.5–34.6 × 3.2–7.2	4.3–5.5 × 2.7–3.3	1.65–1.69	This study
*F. tianshanensis*	Tianshan Mountains of northwestern China	Annual to perennial, effused-reflexed to pileate	Dark bluish gray to yellowish brown	*Picea*	1–3	15.5–44 × 3.3–6.5	6.3–7 × 3.2–3.8	1.9–1.96	This study

In the phylogenetic trees (Figures 1, 2), *Fomitopsis abieticola* is closely related to *F. tianshanensis*. Morphologically, both *F. abieticola* and *F. tianshanensis* have an annual to perennial growth habit, cream to pinkish pore surface when fresh, and large pores, but *F. tianshanensis* differs in its soft corky basidiocarps when fresh, and usually grows on *Picea*. *Fomitopsis schrenkii* has similar pores (3–4 per mm), but it has smaller basidia (12–22 × 6–8 μm), and slightly wider basidiospores (5.7–6.7 × 3.7–4.2 μm). *Fomitopsis hengduanensis* was also discovered in the Yunnan Province, but *F. hengduanensis* differs with smaller pores (6–8 per mm) and smaller basidiospores (5.2–6 × 3.2–3.6 μm).

Phylogenetically, *Fomitopsis hengduanensis* grouped together with *F. kesiyae* (Figures 1, 2). Morphologically, *F. hengduanensis* and *F. kesiyae* share a white to cream pore surface when fresh and have similar pores, but *F. kesiyae* differs in having a buff yellow to orange-yellow buff pileal surface when fresh, reddish brown to yellowish brown when dry, and grows on the *Pinus kesiya* tree. *Fomitopsis pinicola*, *F. schrenkii* and *F. mounceae* all have similar pilei, but they have larger pores (4–6 per mm in *F. pinicola*, 3–4 per mm in *F. schrenkii*, 3–5 per mm in *F. mounceae*; Table 2). *Fomitopsis subpinicola* has similar sized pores, but it has smaller basidia (16.1–24.5 × 4.5–9 μm) and basidiospores (4.3–5.5 × 2.7–3.3 μm) and is distributed in south northeast China.

*Fomitopsis kesiyae* was described from Vietnam on the tree of *Pinus kesiya*. Phylogenetically, two sampled specimens of *F. kesiyae* formed a high supported lineage (99% ML, 99% MP, 1.00 BPP) and are closely related to *F. hengduanensis* (Figures 1, 2). However, *F. hengduanensis* differs in having larger basidia (16.6–34.5 × 5.4–10.2 μm) and basidiospores (5.2–6 × 3.2–3.6 μm). *Fomitopsis subpinicola* has similar sized pores (6–8 per mm) and basidiospores (4.3–5.5 × 2.7–3.3 μm), but it has an apricot-orange, scarlet to fuscous pileal surface when fresh, and is reddish brown to dark brown when dry. *Fomitopsis massoniana* has a similar colored pileal surface when fresh and similar sized pores (5–7 per mm), but it has larger basidiospores (6.2–7.3 × 3.3–4 μm) and grows on *Pinus massoniana* rather than *Pinus kesiya*.

Morphologically, *Fomitopsis massoniana* is similar to *F. kesiyae*; both species have an annual growth habit and a similar colored pileal surface when fresh. However, *F. kesiyae* differs in having smaller basidiospores (4.8–5.3 × 3–3.5 μm), is distributed in Vietnam, and grows on *Pinus kesiya*. *Fomitopsis hengduanensis* has similar sized pores (6–8 per mm), but compared to *F. massoniana*, *F. hengduanensis* has larger sized basidiocarps, a laccate pileal surface with pale dark gray to reddish brown at base and cream to flesh-pink toward the margin when fresh, and smaller sized basidiospores (5.2–6 × 3.2–3.6 μm). Phylogenetically, these two species are distinct from each other (Figures 1, 2). *Fomitopsis massoniana* is closely related to *F. subpinicola*, they share a cream to buff pore surface and have similar sized pores, but *F. subpinicola* has smaller basidiospores (4.3–5.5 × 2.7–3.3 μm).

*Fomitopsis subpinicola* can be easily separated from *F. pinicola* by its apricot-orange, scarlet to fuscous pileal surface when fresh, reddish brown to dark brown when dry, smaller basidiospores (4.3–5.5 × 2.7–3.3 μm) and is located in the Northeast of China. Phylogenetically, *F. subpinicola* is closely related to *F. massoniana*. Morphologically, *F. subpinicola* is similar to *F. massonian*, which has an annual growth habit and white to cream pore surface when fresh. But *F. massoniana* differs by its effused-reflexed to pileate basidiocarps, lager basidiospores (6.2–7.3 × 3.3–4 μm) and grows on the *Pinus massoniana* tree. *Fomitopsis hengduanensis* and *F. kesiyae* have similar sized pores (6–8 per mm), but *F. hengduanensis* has larger sized basidia (16.6–34.5 × 5.4–10.2 μm), *F. kesiyae* has a buff yellow to orange-yellow buff pileal surface when fresh, reddish brown to yellowish brown when dry, and grows on the *Pinus kesiya* tree.

Phylogenetically, specimens of *Fomitopsis tianshanensis* formed a well-supported lineage (Figures 1, 2) and are grouped with *F. abieticola.* But *F. abieticola* differs from *F. tianshanensis* in having large sized cystidioles (17.5–50.2 × 4.3–9.5 μm), basidia (20.8–40.5 × 5.5–11.5 μm), and basidiospores (7–9 × 4–5 μm). *Fomitopsis pinicola* also grows mainly on *Picea*, but it has smaller pores (4–6 per mm), a brownish orange to black pileal surface at base and buff-yellow to cinnamon toward the margin when fresh, and is distributed in Europe.

Previously, *Fomitopsis pinicola* was used as a broad species concept, specimens from Europe, North America, and East Asia were all identified as *F. pinicola* based on morphological characters (Gilbertson and Ryvarden, 1986; Ryvarden and Gilbertson, 1993; Núñez and Ryvarden, 2001; Dai, 2012; Ryvarden and Melo, 2014). Recent phylogenetic analyses indicated that *F. pinicola* is a species complex and represent several different species. Haight et al. (2016) proposed four well-supported clades: one lineage *F. pinicola* sensu stricto from Europe and three lineages from North America. Subsequently, Haight et al. (2019) identified these three lineages as *F. mounceae*, *F. ochracea*, and *F. schrenkii* from North America, and *F. pinicola* is restricted to Eurasia and does not occur in North America. In the current study, samples previously identified as *F. pinicola* from China and Vietnam in East Asia represent six distinct species. Furthermore, our results indicated that species of the *F. pinicola* complex usually have limited distribution areas and host specialization. In East Asia, *F. abieticola* is distributed in southwestern China and grows on *Abies*; *F. hengduanensis* is distributed in high altitude areas of the Hengduan Mountains of southwestern China, and it mostly grows on *Picea*, occasionally on other gymnosperm wood; *F. kesiyae* is distributed in tropical areas of Vietnam and grows only on *Pinus kesiya*; *F. massoniana* is distributed in southeastern China and grows only on *Pinus massoniana*; *F. subpinicola* was found in northeastern China and grows mainly on *Pinus koraiensis*, occasionally on other gymnosperm or angiosperm wood; *F. tianshanensis* is distributed in Tianshan Mountains of northwestern China and only grows on *Picea schrenkiana*. In Europe, *F. pinicola* is widespread, and it mostly grows on *Picea* and *Pinus*, occasionally on other different gymnosperm or angiosperm wood (Ryvarden and Melo, 2014). In North America, *F. mounceae* and *F. ochracea* is distributed in Canada and the northern United States, and they grow on different gymnosperm or angiosperm wood (Ryvarden and Stokland, 2008; Haight et al., 2019); *F. schrenkii* is distributed in western and southwestern regions of the United States and mostly grows on different gymnosperm wood, rarely on angiosperm wood (Haight et al., 2019). Among the wood-rotting fungi, some other polypore genera also have limited distribution areas and host specializations, such as *Bondarzewia* Singer (Song et al., 2016), *Heterobasidion* Bref. (Chen et al., 2015; Yuan et al., 2020), *Laetiporus* Murrill (Song and Cui, 2017), and *Sanghuangporus* Sheng H. Wu, L.W. Zhou & Y.C. Dai (Zhu et al., 2019).

### Key to Accepted Species of *Fomitopsis pinicola* Complex

**Table:** 

1.	Distribution in East Asia	2
1.	Distribution in North America or Europe	7
2.	Pores < 5 per mm	3
2.	Pores 5–8 per mm	4
3.	Basidiospores oblong-ellipsoid to ellipsoid, 7–9 × 4–5 μm	*F. abieticola*
3.	Basidiospores oblong-ellipsoid, 6.3–7 × 3.2–3.8 μm	*F. tianshanensis*
4.	Distribution in tropical areas	*F. kesiyae*
4.	Distribution in temperate areas	5
5.	Basidiocarps annual to perennial; pileal surface cream to flesh-pink toward the margin when fresh	*F. hengduanensis*
5.	Basidiocarps annual; pileal surface white to cream toward the margin when fresh	6
6.	Basidiospores oblong-ellipsoid, 6.2–7.3 × 3.3–4 μm	*F. massoniana*
6.	Basidiospores oblong-ellipsoid to ellipsoid, 4.3–5.5 × 2.7–3.3 μm	*F. subpinicola*
7.	Pilei never with reddish brown band	*F. ochracea*
7.	Pilei with reddish brown band	8
8.	Distribution in Europe; basidia < 20 μm long	*F. pinicola*
8.	Distribution in North America; basidia > 20 μm long	9
9.	Basidiospores ellipsoid to cylindrical, Q = 1.6–1.9	*F. mounceae*
9.	Basidiospores ellipsoid to broadly cylindrical, Q = 1.5–1.6	*F. schrenkii*

## Data Availability Statement

The datasets presented in this study can be found in an online repository. The name of the repository and accession number can be found below: https://treebase.org/treebase-web/search/study/summary.html?id=27994&x-access-code=ab2495717aaf081f0557e6680c381710&agreement=ok, submission ID: 27994.

## Author Contributions

B-KC and SL designed the experiment. SL, M-LH, D-MW, and B-KC prepared the samples. SL, T-MX, and YW conducted the molecular experiments and analyzed the data. SL, D-MW, and B-KC drafted the manuscript. All the authors approved the manuscript.

## Conflict of Interest

The authors declare that the research was conducted in the absence of any commercial or financial relationships that could be construed as a potential conflict of interest.

## Footnotes

1 http://mafft.cbrc.jp/alignment/server/

2 http://www.treebase.org

3 http://tree.bio.ed.ac.uk/software/figtree/

## Abbreviations

BI: Bayesian inference
BJFC: Herbarium of the Institute of Microbiology, Beijing Forestry University
BGI: Beijing Genomics Institute
BPP: Bayesian posterior probabilities
BT: Bootstrap
CB: Cotton Blue
CB–: acyanophilous
GTR + I + G: General time reversible + proportion invariant + gamma
IKI: Melzer’s reagent
IKI–: neither amyloid nor dextrinoid
ILD: incongruence length difference test
ITS: internal transcribed spacer
KOH: 5% potassium hydroxide
L: mean spore length (arithmetic average of all spores)
ML: maximum likelihood
MP: maximum parsimony
MPT: most parsimonious tree
n (a/b): number of spores (a) measured from given number (b) of specimens
Q: variation in the L/W ratios between the specimens studied
RPB2: DNA-directed RNA polymerase II subunit 2
TL: tree length
W: mean spore width (arithmetic average of all spores)
CI: consistency index
RI: retention index
RC: rescaled consistency index
HI: homoplasy index
TEF: translation elongation factor 1-α.
